# Overlapping group screening for detection of gene-gene interactions: application to gene expression profiles with survival trait

**DOI:** 10.1186/s12859-018-2372-2

**Published:** 2018-09-21

**Authors:** Jie-Huei Wang, Yi-Hau Chen

**Affiliations:** Institute of Statistical Science, Academia Sinica, Nankang, Taipei, Taiwan

**Keywords:** Gene-gene interaction, Lasso, Overlapping group, Survival prediction

## Abstract

**Background:**

The development of a disease is a complex process that may result from joint effects of multiple genes. In this article, we propose the overlapping group screening (OGS) approach to determining active genes and gene-gene interactions incorporating prior pathway information. The OGS method is developed to overcome the challenges in genome-wide data analysis that the number of the genes and gene-gene interactions is far greater than the sample size, and the pathways generally overlap with one another. The OGS method is further proposed for patients’ survival prediction based on gene expression data.

**Results:**

Simulation studies demonstrate that the performance of the OGS approach in identifying the true main and interaction effects is good and the survival prediction accuracy of OGS with the Lasso penalty is better than the ordinary Lasso method. In real data analysis, we identify several significant genes and/or epistasis interactions that are associated with clinical survival outcomes of diffuse large B-cell lymphoma (DLBCL) and non-small-cell lung cancer (NSCLC) by utilizing prior pathway information from the KEGG pathway and the GO biological process databases, respectively.

**Conclusions:**

The OGS approach is useful for selecting important genes and epistasis interactions in the ultra-high dimensional feature space. The prediction ability of OGS with the Lasso penalty is better than existing methods. The OGS approach is generally applicable to various types of outcome data (quantitative, qualitative, censored event time data) and regression models (e.g. linear, logistic, and Cox’s regression models).

**Electronic supplementary material:**

The online version of this article (10.1186/s12859-018-2372-2) contains supplementary material, which is available to authorized users.

## Background

Discovering important pathways, genes, and gene-gene interactions that account for the phenotype of interest has continued to be a key challenge in genome-wide expression analysis [1]. Under this high-dimensional data setting, single and multiple biomarker (e.g. gene) tests commonly used usually have limited power to detect causal biomarkers associated with the clinical phenotypes. To improve the power, analyses incorporating external biological information have been proposed. For example, gene-based analyses group the single-nucleotide polymorphisms (SNPs) under study into genes, and pathway-based analyses group the genes under study into some biologically meaningful pathways; both types of multiple biomarker analyses have shown to be effective in detecting causal association signals and become increasingly popular. The analyses incorporating external biological information are particularly useful for detecting interaction effects among biomarkers, since the number of interaction effects grows quickly with the number of biomarkers and hence traditional statistical tests lose power.

To identify causal interaction effects of single-nucleotide polymorphisms (SNPs) on a quantitative or disease trait, Fang et al. [2] develop a two-stage grouped sure independence screening (TS-GSIS) procedure using gene-based SNP sets. Simulation studies demonstrate that the performance of TS-GSIS is better than some existing approaches, including the extended SVM [3] and the TS-SIS method [4] without incorporating gene set information. A potential drawback for the TS-GSIS method is that, it is developed in the setting where the groups (gene sets) are non-overlapping and does not pay attention to settings with overlapping groups, which would be encountered in pathway-based analyses where different pathways may involve some common genes. Besides, TS-GSIS is focused specifically on the quantitative/qualitative outcome modeled by linear/logistic regression, and its application to the survival outcome has not been examined.

In this work, we propose the overlapping group screening (OGS) method, which is an extension and improvement of TS-GSIS to accommodate overlapping group structures. Following the latent effect approach of Jacob et al. [5], we decompose the original biomarker effects into a sum of group-specific latent effects, so that the original overlapping group structure can be transformed into a new non-overlapping group structure. The latent effect approach has also been applied by Zeng and Breheny [6], Zhang et al. [7] and Tang et al. [8] to joint selection of genes and genetic pathways.

In addition, to perform association analyses with general types of traits including survival endpoints, OGS employs the sequence kernel association test (SKAT) proposed by Chen et al. [9] as the group screening criterion. SKAT is a supervised, flexible, and computationally efficient regression method to test for association between genetic variants/gene expressions in a region and a quantitative/qualitative/survival trait [10]. In particular, SKAT can quickly compute *p*-values analytically by fitting the null model only once, and hence can be conveniently applied to genome-wide data. Further, we utilize a data-driven thresholding strategy of Fan et al. [11] for screening candidate biomarkers/features, where we permute randomly the original biomarker data among subjects to decouple the association between the biomarker and outcome data, such that the permuted data follow the null distribution, from which a cut-off value for the SKAT *p*-value to determine significance can be determined. After screening candidate biomarkers by the SKAT *p*-values, we apply the Ridge or Lasso penalized regression method [12] to build the prediction model in OGS. The Lasso penalty, in particular, allows for automatic variable selection, which are commonly employed in high-dimensional data such as genome-wide data analysis.

We note that OGS maintains the advantages of TS-GSIS, namely: (i) it can mitigate the issue of co-linearity in regression analyses owing to correlations among biomarkers in the same gene/pathway, and (ii) it can substantially reduce the search space for interaction effects by utilizing the feature grouping structure.

The other objective of this article is to predict survival outcomes based on gene expression profiles, a topic which has received much attention in the recent decade ([13–15] and so on). Zhang et al. [16] indicate that one of the main shortcomings of the past studies is the failure to incorporate prior biological information into the prediction model, which may in turn lead to inaccurate prognosis and prediction. The survival prediction based on OGS addresses this problem. Simulation studies demonstrate that the OGS approach not only identifies correctly the causal biological pathways and epistasis, but also improves survival prediction compared with the alternative analyses that ignore the pathway information.

In the real data application, we utilize OGS to select several causal genes and epistasis that are associated with clinical survival outcomes of diffuse large B-cell lymphoma (DLBCL) and non-small-cell lung cancer (NSCLC) patients. In these applications, we combine gene expression profile data with prior pathway information from the KEGG pathway database (for DLBCL) and the Gene Ontology (GO) biological process database (for NSCLC), which are popular public databases providing information on discovered pathways and their involved genes [17]. We use the pathway information available from these two databases to assign genes into groups based on the specific pathways in which they are involved, and conduct survival prediction based on the selected genes and gene-gene interactions.

## Motivation

**Fig. 1:**
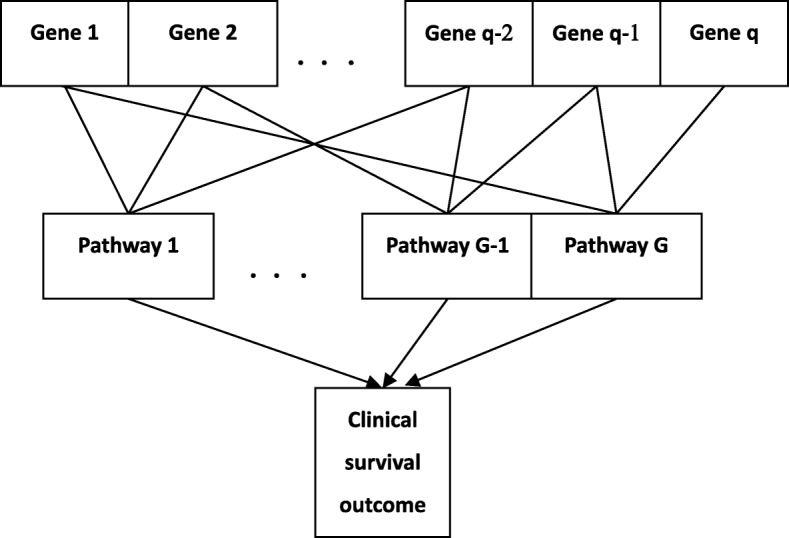
The natural hierarchal structure of genes related to pathways with the clinical outcome

Suppose that there are q genes assigned to G possibly overlapping pathways, namely, a given gene may belong to more than one pathway. The schematic plot in Fig. 1 displays the natural hierarchal structure of genes related to pathways and shows the overlapping pathway structure present in the gene expression data. Each gene can belong to one or multiple pathways. It is of interest to identify genes, as well as their interactions, that are associated with the clinical survival outcome.

### Survival model

Let **X** denote the *N* × *q* dimensional covariate matrix of the gene expression profiles with $$ \mathrm{X}={\left({\mathrm{x}}_1,\mathrm{L},{\mathrm{x}}_N\right)}^{/}={\left(\begin{array}{ccc}{x}_{11}& \cdots & {x}_{1q}\\ {}\vdots & \ddots & \vdots \\ {}{x}_{N1}& \cdots & {x}_{Nq}\end{array}\right)}_{N\times q} $$, where *x*_*ij*_ denotes the expression level of the *j-th* gene of the *i-th* subject. Assume the survival outcome *T*_*i*_ is related to the gene expression covariates **x**_*i*_ through a Cox’s regression model. In the Cox’s regression framework, the hazard function at time *t* for subject *i*‘s survival given the covariates is modeled as

$$ \lambda \left(t|{\mathbf{x}}_i\right)={\lambda}_0(t)\exp \left({\mathbf{x}}_i^{/}\boldsymbol{\upbeta} \right), $$

where *λ*_0_(*t*) is a non-negative deterministic baseline hazard function and **β** = (*β*_1_, ⋯, *β*_*q*_)^/^ is the logarithm of the risk ratio. Based on the Cox’s model, the survival function of subject *i* given his/her expression profile **x**_*i*_ is given by $P\left({T}_i>t|{\mathbf{x}}_i\right)=S\left(t|{\mathbf{x}}_i\right)={S}_0{(t)}^{\exp \left({\mathbf{x}}_i^{/}\boldsymbol{\upbeta} \right)}$ with ${S}_0(t)=\exp \left[-{\int}_0^t{\lambda}_0(s) ds\right]$ the baseline survival. Usually the survival outcome is subject to censoring, and we use ${t}_i^{\ast }$ to denote the observed survival time of subject *i*, and ${\delta}_i^{\ast }$ is the indicator of whether the survival time of subject *i* is censored.

In practice, we can check the Cox’s model assumption by existing approaches, such as statistical tests and graphical diagnostics based on the Schoenfeld residuals [18].

### Latent effect approach

Incorporating the grouping (pathway) information into the modeling process has the potential to improve the interpretability and the accuracy of the model. When the groups overlap one another, special techniques are required to adequately account for the overlapping grouping information. According to Jacob et al. [5], we decompose the original coefficient vector into a sum of group-specific latent effects, namely, $\boldsymbol{\upbeta} =\sum \limits_{j=1}^G{\boldsymbol{\upgamma}}^j$, where ${\boldsymbol{\upgamma}}^j={\left({\gamma}_1^j,\mathrm{L},{\gamma}_q^j\right)}^{/}$ is the latent coefficient vector for group *j*. Here is a simple example for illustration [6]. Suppose that there are four genes that are involved in the four pathways, P1 = {g1, g2}, P2 = {g2, g3}, P3 = {g1, g3} and P4 = {g3, g4}, the original coefficient **β** can be decomposed as **β** = [*β*_1_, *β*_2_, *β*_3_, *β*_4_]^/^

$$ =\left[\begin{array}{l}{\gamma}_1^1\\ {}{\gamma}_2^1\\ {}{\gamma}_3^1\\ {}{\gamma}_4^1\end{array}\right]+\left[\begin{array}{l}{\gamma}_1^2\\ {}{\gamma}_2^2\\ {}{\gamma}_3^2\\ {}{\gamma}_4^2\end{array}\right]+\left[\begin{array}{l}{\gamma}_1^3\\ {}{\gamma}_2^3\\ {}{\gamma}_3^3\\ {}{\gamma}_4^3\end{array}\right]+\left[\begin{array}{l}{\gamma}_1^4\\ {}{\gamma}_2^4\\ {}{\gamma}_3^4\\ {}{\gamma}_4^4\end{array}\right] $$ $$ =\left[\begin{array}{l}{\gamma}_1^1\\ {}{\gamma}_2^1\\ {}0\\ {}0\end{array}\right]+\left[\begin{array}{l}0\\ {}{\gamma}_2^2\\ {}{\gamma}_3^2\\ {}0\end{array}\right]+\left[\begin{array}{l}{\gamma}_1^3\\ {}0\\ {}{\gamma}_3^3\\ {}0\end{array}\right]+\left[\begin{array}{l}0\\ {}0\\ {}{\gamma}_3^4\\ {}{\gamma}_4^4\end{array}\right] $$

$$ =\left[\begin{array}{l}1,0,0,0,1,0,0,0\\ {}0,1,1,0,0,0,0,0\\ {}0,0,0,1,0,1,1,0\\ {}0,0,0,0,0,0,0,1\end{array}\right]{\left[{\gamma}_1^1,{\gamma}_2^1,{\gamma}_2^2,{\gamma}_3^2,{\gamma}_1^3,{\gamma}_3^3,{\gamma}_3^4,{\gamma}_4^4\right]}^{/} $$

$$ =\mathbf{S}\boldsymbol{\upgamma } . $$

Based on the coefficient decomposition, the original regression model can be transformed into a new model, i.e. ${\mathbf{X}}_{N\times q}{\boldsymbol{\upbeta}}_{q\times 1}={\mathbf{X}}_{N\times q}{\mathbf{S}}_{q\times u}{\boldsymbol{\upgamma}}_{u\times 1}={\overset{\sim }{\mathbf{X}}}_{N\times u}{\boldsymbol{\upgamma}}_{u\times 1}$. Equivalently, this new model can be constructed by duplicating the columns of overlapped variables in the raw design matrix. For the new transformed model, the hazard function for subject *i* in the Cox’s regression model is re-expressed as

$$ \lambda \left(t|{\overset{\sim }{\mathbf{x}}}_i\right)={\lambda}_0(t)\exp \left({\overset{\sim }{\mathbf{x}}}_i^{/}\boldsymbol{\upgamma} \right). $$

### Method (OGS)

We propose the OGS method and apply it to the gene expression profile data with clinical survival trait to detect causal genes and epistasis interactions by incorporating prior pathway information. We standardized all the predictors before performing the OGS approach. The steps of the OGS algorithm are described as follows.

Step1: Based on the latent effect approach, we utilize the overlapping group Cox’s regression model to identify the causal pathways, which can be computed by the R package “grpregOverlap” [6]. We define ${\hat{\mathrm{M}}}_{main}$ as the selected set of causal pathways, and $A=\left|{\hat{\mathrm{M}}}_{main}\right|$ as the size of ${\hat{\mathrm{M}}}_{main}$.

Step 2: Consider gene-gene interaction pairs between gene pairs from one causal pathway or two different causal pathways in ${\hat{\mathrm{M}}}_{main}$, as well as gene pairs between one pathway in ${\hat{\mathrm{M}}}_{main}$ and one non-causal pathway outside ${\hat{\mathrm{M}}}_{main}$. The interaction between two pathways is also termed “cross-talk” of pathways [19]. For groups of gene-gene interaction pairs from each of the candidate pathways or from each two cross-talk pathways, apply the SKAT test to obtain the group-specific significance. Detail about the group-specific SKAT test is given in the next section.

Step 3: We randomly permute the original genotype matrix **x**_*i*_ to form the permuted data {Y_*i*_, **x**_*π*(*i*)_} following the null model, where {*π*(1), ⋯, *π*(*N*)} is a random permutation of the index. Then apply again the SKAT test for each of the pathway interaction groups with the permuted data to obtain the group screening measures (*p*-values) $\left\{{p}_1^{\ast },\cdots, {p}_B^{\ast}\right\}$. We adopt ${C}_{\mathrm{int}}=\min \left\{{p}_1^{\ast },\cdots, {p}_B^{\ast}\right\}$ as a cutoff point to select candidate pathway interactions, i.e.

$$ {\hat{\mathrm{M}}}_{\mathrm{int}}=\left\{b:{p}_b<{C}_{\mathrm{int}},b=1,\cdots, B\right\}, $$

is our selected set of pathway interactions.

Step 4: Apply the penalized Cox’s regression with the Ridge, or Lasso penalty to build the final prediction model based on genes in ${\hat{\mathrm{M}}}_{main}$ and gene-pair interactions in ${\hat{\mathrm{M}}}_{\mathrm{int}}$. Note that when applying the Lasso penalty, some of the genes/gene pairs in ${\hat{\mathrm{M}}}_{main}$/${\hat{\mathrm{M}}}_{\mathrm{int}}$ may be removed since the Lasso penalty can set some of the coefficients exactly to 0, while when applying the Ridge penalty, all of the candidate genes and gene pairs are retained. The penalized Cox’s model with the Ridge and Lasso penalties can be obtained by the R package “*glmnet”* [12].

### Group-specific test (SKAT)

Following Chen et al. [9], the group-specific SKAT statistic under the Cox’s regression model is given as

$$ {Q}_{(k)}={\mathbf{m}}^{/}{\mathbf{R}}_{(k)}{\mathbf{W}}_{(k)}{\mathbf{W}}_{(k)}{\mathbf{R}}_{(k)}^{/}\mathbf{m},k=1,\cdots, \mathrm{B} $$

Here, $B=A+{C}_2^A+\left(G-A\right)\times A$ is the total number of groups of pathway interaction, **m** is the vector of martingale residuals estimated from the null model without considering the gene expression data, **R**_(*k*)_ = [*r*_(*k*)*ij*_]_*N* × *l*_,

where *l* is the number of gene-gene interaction pairs in the pathway interaction group k, *r*_(*k*)*ij*_ is the *j-th* gene-gene interaction pair of *i-th* subject in the pathway interaction group k, and **W**_(*k*)_ is a diagonal weight matrix that contains the weights of the *l* interaction pairs in the pathway interaction group k. Suitable weights can improve the testing power [10]. We utilize the penalized Cox’s partial likelihood approach with the Ridge penalty to estimate effect sizes for gene-gene interaction pairs in each pathway interaction group, and take the square root of the absolute estimated coefficients as our weights, i.e.,

$$ {\mathbf{W}}_{(k)}={\left(\begin{array}{ccc}\sqrt{\left|{\overset{\sim }{\boldsymbol{\upbeta}}}_{(k)1}\right|}& & 0\\ {}& \ddots & \\ {}0& & \sqrt{\left|{\overset{\sim }{\boldsymbol{\upbeta}}}_{(k)l}\right|}\end{array}\right)}_{l\times l}, $$

Based on null model without gene covariates, let **V** =  *diag* (e_1_, …, e_*N*_) − **PP**^/^, where **P** is an *N* × *v* matrix with element *p*_*ij*_the baseline hazard for individual *i* at ordered failure time ${t}_{(j)}^{\ast },j=1,\cdots, v$, and e_*i*_ the cumulative hazard for individual *i* at observed time ${t}_i^{\ast }$. Let ${\Sigma}_{(k)}={\mathbf{W}}_{(k)}{\mathbf{R}}_{(k)}^{/}{\mathbf{VR}}_{(k)}{\mathbf{W}}_{(k)}$ be the covariance matrix of the vector ${\mathbf{W}}_{(k)}{\mathbf{R}}_{(k)}^{/}\mathbf{m}$ under the null hypothesis of all gene-gene interaction pairs in the pathway interaction group k having null effects. Under the null hypothesis, the SKAT statistic follows a mixture chi-square distribution:

$$ {Q}_{(k)}\sim \sum \limits_{j=1}^l{\lambda}_{(k)j}{\chi}_{1,j}^2, $$

where *λ*_(*k*)*j*_, *j* = 1, ⋯, *l* are the eigenvalues of Σ_(*k*)_, and ${\chi}_{1,j}^2$‘s are independent 1-df central chi-square random variables.

We use the Davies method [20] to approximate the tail probability (*p*-value) of the mixture chi-square distributions, which can be computed by R package “*CompQuadForm*” [21]. In general, the Davies method is accurate [22]. The *p*-values {*p*_1_, ⋯, *p*_*B*_} for the pathway interaction groups serve as our group screening measures; a smaller p-value corresponds to higher significance of the group and hence leads to higher priority to be selected.

## Results

In the following simulations, we investigate the performances of the proposed OGS approach in variable selection, estimation, and prediction, and compared them with those from the “Oracle”, “Univariate Selection”, “Ordinary Lasso”, and “TS-GSIS Lasso” methods. The “Oracle” method is based on the underlying true model, which is known in simulations but unknown in real applications. The “Univariate Selection” method selects the genes and gene-pairs one by one via univariate regression, with controlled false discovery rate (< 0.05), and the selected variables are included in a multivariate Cox’s regression model to form the final prediction model. The “Ordinary Lasso” method is the penalized Cox’s regression model with the covariates of gene expressions from all genes and gene-pair interactions and with the Lasso penalty. The “TS-GSIS Lasso” method is essentially proposed by Fang et al. [2], except that we apply the SKAT test to obtain the group-specific significance.

For performance comparison, we obtain the root mean squared error (RMSE) to measure estimation accuracy, defined as

$$ RMSE=\sqrt{\frac{1}{S}\sum \limits_{j=1}^S{\left({\beta}_j-{\hat{\beta}}_j\right)}^2}, $$

where *S* is full model size including all main and interaction covariates. Over 500 simulations, we report the median value RMSE.M of RMSE over simulations. We also report the following proportions in 500 simulations as performance measures for variable selection: T.model is the proportion where the selected model includes the underlying effective variables, including both the main and interaction terms; Tint.model is the proportion where the selected model includes the underlying effective gene-gene interaction terms; Sen. is the sensitivity, i.e., the proportion of the underlying effective variables being selected; Spe. is the specificity, i.e., the proportion of the underlying ineffective variables not being selected. We also report the median size S.model of the selected model over 500 simulations. For assessing the performance in survival prediction, we report two measures of prediction accuracy: the deviance and *c*-index proposed by Harrell et al. [23] and smaller deviance/larger *c*-index corresponds to better prediction accuracy. The median values of deviance and *c*-index over 500 simulations are reported.

Also, let $\hat{\boldsymbol{\upbeta}}$ be an estimator of the (penalized) Cox’s regression parameter in a prediction model obtained from the training dataset and $\left({t}_i^{\ast },{\delta}_i^{\ast },{\mathbf{x}}_i^{\ast}\right)$ the survival and covariate data of subject *i* in the test data. Define ${\mathbf{x}}_i^{\ast}\hat{\boldsymbol{\upbeta}}$ as the prognosis index (PI) value for subject *i*. The prediction accuracy measure of Cox-test is defined as the *p*-value of PI when PI is used as the covariate in the univariate Cox model for the survival outcome in the test data. A smaller value of Cox-test (p-value) would suggest better prediction accuracy. Similarly, the prediction accuracy measure of LR-test is the p-value of the log-rank test for the null hypothesis of equality of the survival between the “poor” and “good” prognosis groups in the test data, which are formed according to whether the PI value is higher or lower than the median PI value. A smaller LR-test value corresponds to better prediction power.

We consider survival data with a cohort size 500 as the training set, where each subject’s survival time follows the Cox’s proportional hazards model

$$ {\lambda}_0\left(\left.t\right|\mathbf{x}\right)=0.1\cdot \exp \left({\mathbf{x}}^{/}\boldsymbol{\upbeta} \right), $$

with **β** measuring the log-relative risk with respect to the covariates and the covariates **x** jointly following a multivariate standard normal distribution with correlation *corr*(*x*_⋅*j*_, *x*_⋅*k*_) = 0.5^|*j* − *k*|^. The censoring time distribution follows a uniform *U*(0, 1) distribution. We then generate survival data, independent of the training data, with a cohort of size 100 as the test data to assess the prediction accuracy for different methods.

### Simulation setting 1

In this simulation study, the design matrix consists of 5 groups with each group having different group sizes. The group size (number of genes in each pathway) and the overlapping structure (number of genes shared by two overlapping pathways) are shown  in Table 1.

**Table 1:** Data structure in Simulation 1

Pathway	1		2		3		4		5
Gene Size	7		14		21		28		35
Overlapping		3		5		7		9	

**Fig. 2:**
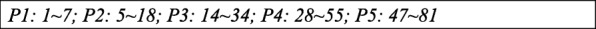
The gene indices of the pathways considered in Simulation 1

For example, pathways 1 and 2 contain 7 and 14 genes, respectively. The two groups contain 18 unique genes, and 3 genes are shared by the two groups. As a result, there are 81 genes (q = 81) and 105 latent effects in this example. Fig. 2 shows the gene indices of the pathways. Pathways 2 and 4 are effective, and genes in each of them have constant latent effects of 4.5 and − 3, respectively. Three types of gene-gene interactions are considered: (1) gene-gene interactions (*x*_⋅8_ × *x*_⋅9_, *x*_⋅10_ × *x*_⋅11_, *x*_⋅12_ × *x*_⋅13_) within pathway 2 with effects(6, 6, 6), (2) gene-gene interaction (*x*_⋅36_ × *x*_⋅66_, *x*_⋅38_ × *x*_⋅68_, *x*_⋅40_ × *x*_⋅70_) across pathways 4 and 5 with effects(−6, −6, −6), and (3) coexistence of interactions (1) and (2). The number of effective genes and gene-pair interactions is 45 or 48 among the total 3321 genes and gene-pairs. We examine performances of different methods under a censoring rate of 50% or 65%.

### Simulation setting 2

In this simulation study, the design matrix consists of 24 groups with each group having different group sizes, ranging from 3 to 60 (genes). The group size and the overlapping structure are shown in Table 2.

**Table 2:** Data structure in Simulation 2

Pathway	1	2	3	4	5	6	7	8	9	10	11	12	13	14	15	16	17	18	19	20	21	22	23	24
Gene Size	3	3	3	6	6	6	9	9	9	15	15	15	24	24	24	36	36	36	45	45	45	60	60	60
Overlapping	1 1			2 2			3 3			5 5			8 8			12 12			15 15			20 20		

**Fig. 3:**
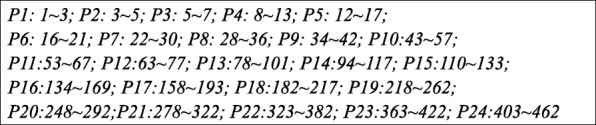
The gene indices of the pathways considered in Simulation 2

For example, pathway 4 contains 6 genes, as group 5 does, and the two groups contain 10 unique genes, and 2 genes are shared by the two groups. As a result, there are 462 genes (q = 462) and 594 latent effects in this example. Fig. 3 shows the gene indices of the pathways. Pathways 1, 7, 13, and 19 are effective, and genes in each of them have constant latent effects of 4.5, − 3, − 3, and 1.5, respectively. As above, three types of gene-gene interactions are considered: (1) gene-gene interactions (*x*_⋅22_ × *x*_⋅23_, *x*_⋅24_ × *x*_⋅25_, *x*_⋅26_ × *x*_⋅27_) within pathway 7 with effects(4, 4, 4), (2) gene-gene interaction (*x*_⋅81_ × *x*_⋅101_, *x*_⋅82_ × *x*_⋅102_, *x*_⋅83_ × *x*_⋅103_) across pathways 13 and 14 with effects(−4, −4, −4), and (3) coexistence of interactions (1) and (2). The number of effective genes and gene-pair interactions is 84 or 87 among the total 106,953 genes and gene-pairs. We examine different methods under a censoring rate of 50% or 65%.

### Summary of simulation results

From the simulation results shown in Tables 3, 4, 5, 6, 7, and 8, the OGS method using the Lasso penalty outperforms the OGS method using the Ridge penalty. Also, compared to the existing methods, OGS with the Lasso penalty performs substantially better than the Univariate Selection and the TS-GSIS with the Lasso penalty methods in variable selection (T.model, Tint.model, Sen., Spe.), estimation (RMSE.M), and prediction (Deviance, *c*-index). When the number of groups (pathways) and the group size (number of genes) are smaller (Setting 1) and the censoring rate is relatively lower (50%), the ordinary Lasso also performs well in variable selection and survival prediction; while in other cases, the ordinary Lasso is less competitive than the proposed OGS method with the Lasso penalty in variable selection, estimation, and survival prediction. Comparing Tables 3, 4, and 5, or Tables 6, 7, and 8, we see that the pattern of interactions, namely whether the gene-gene interactions occur within the same pathway or not, does not affect much the performance of the proposed OGS method, in particular for survival prediction.

**Table 3:** Results of Simulation 1 (1): The performances of OGS compared with other approaches under gene-gene interactions within one pathway

	Oracle	Uni. Sel.	Ordinary Lasso	TS-GSIS Lasso	OGS Ridge	OGS Lasso
censoring rate = 50%						
RMSE.M	0.422	0.425	0.355	0.340	0.435	0.325
T.model	1	0	1	0.625	0.650	0.650
Tint.model	1	0.075	1	0.625	0.650	0.650
Sen.	1	0.783	1	0.974	0.977	0.976
Spe.	1	0.999	0.954	0.964	0.775	0.970
S.model	45	37.895	197.165	160.375	781.805	142.245
Deviance	− 128.514	− 108.289	− 281.603	− 282.783	− 50.658	− 294.313
* c*-index	0.925	0.891	0.984	0.983	0.855	0.985
censoring rate = 65%						
RMSE.M	0.421	0.424	0.375	0.364	0.436	0.348
T.model	1	0	1	0.805	0.815	0.815
Tint.model	1	0.070	1	0.805	0.815	0.815
Sen.	1	0.764	1	0.986	0.988	0.987
Spe.	1	0.999	0.962	0.965	0.686	0.968
S.model	45	37.855	170.655	157.450	1072.52	148.745
Deviance	−123.803	−102.527	−231.398	− 240.500	− 45.181	−250.026
* c*-index	0.928	0.898	0.983	0.984	0.849	0.986

**Table 4:** Results of Simulation 1 (2): The performances of OGS compared with other approaches under gene-gene interactions across two pathways

	Oracle	Uni. Sel.	Ordinary Lasso	TS-GSIS Lasso	OGS Ridge	OGS Lasso
censoring rate = 50%						
RMSE.M	0.418	0.424	0.382	0.361	0.436	0.349
T.model	1	0	1	0.905	0.915	0.915
Tint.model	1	0.035	1	0.905	0.915	0.915
Sen.	1	0.746	1	0.992	0.994	0.994
Spe.	1	0.999	0.963	0.965	0.666	0.966
S.model	45	36.480	165.850	158.610	1139.54	155.235
Deviance	− 133.967	−102.900	− 219.334	− 241.541	− 42.702	− 252.120
* c*-index	0.944	0.899	0.980	0.984	0.842	0.986
censoring rate = 65%						
RMSE.M	0.414	0.422	0.398	0.390	0.436	0.382
T.model	1	0	0.974	0.909	0.909	0.909
Tint.model	1	0.035	1	0.909	0.909	0.909
Sen.	1	0.729	0.999	0.992	0.994	0.992
Spe.	1	0.999	0.968	0.970	0.558	0.971
S.model	45	36.970	150.597	141.459	1494.17	140.481
Deviance	− 127.447	−92.867	− 169.725	− 182.585	−39.018	− 191.849
* c*-index	0.949	0.903	0.974	0.979	0.829	0.981

**Table 5:** Results of Simulation 1 (3): The performances of OGS compared with other approaches under coexistence of within- and between-pathway gene-gene interactions

	Oracle	Uni. Sel.	Ordinary Lasso	TS-GSIS Lasso	OGS Ridge	OGS Lasso
censoring rate = 50%						
RMSE.M	0.457	0.463	0.400	0.430	0.471	0.393
T.model	1	0	1	0.500	0.525	0.525
Tint.model	1	0	1	0.500	0.525	0.525
Sen.	1	0.708	1	0.959	0.966	0.963
Spe.	1	0.999	0.956	0.968	0.672	0.969
S.model	48	36.970	191.050	151.135	1119.675	146.475
Deviance	−127.602	−95.760	− 266.727	− 206.468	−46.208	− 254.344
* c*-index	0.924	0.871	0.983	0.961	0.826	0.978
censoring rate = 65%						
RMSE.M	0.455	0.461	0.418	0.412	0.471	0.401
T.model	1	0	1	0.675	0.715	0.715
Tint.model	1	0	1	0.675	0.715	0.715
Sen.	1	0.694	1	0.973	0.980	0.977
Spe.	1	0.999	0.963	0.968	0.614	0.969
S.model	48	37.070	168.335	150.420	1308.87	147.785
Deviance	− 126.277	−92.721	− 220.297	− 223.262	−43.936	−235.996
* c*-index	0.929	0.878	0.979	0.978	0.831	0.982

**Table 6:** Results of Simulation 2 (1): The performances of OGS compared with other approaches under gene-gene interactions within one pathway

	Oracle	Uni. Sel.	Ordinary Lasso	TS-GSIS Lasso	OGS Ridge	OGS Lasso
censoring rate = 50%						
RMSE.M	0.065	0.067	0.067	0.067	0.069	0.066
T.model	1	0	0	0.005	0.435	0
Tint.model	1	0	0.545	0.360	0.435	0.415
Sen.	1	0.213	0.592	0.660	0.980	0.722
Spe.	1	1	0.999	0.999	0.760	0.999
S.model	84	19.275	152.135	151.280	25,704	159.140
Deviance	− 136.422	−44.754	−73.930	−88.464	−4.454	−100.153
* c*-index	0.917	0.766	0.853	0.868	0.583	0.885
censoring rate = 65%						
RMSE.M	0.064	0.067	0.067	0.067	0.069	0.067
T.model	1	0	0	0	0.560	0.005
Tint.model	1	0	0.420	0.410	0.560	0.505
Sen.	1	0.204	0.511	0.600	0.984	0.660
Spe.	1	1	0.999	0.999	0.761	0.999
S.model	84	19.095	141.940	141.745	25,586	149.070
Deviance	−128.513	−39.108	−59.558	−74.966	− 12.148	−85.540
* c*-index	0.925	0.769	0.842	0.860	0.605	0.877

**Table 7:** Results of Simulation 2 (3): The performances of OGS compared with other approaches under gene-gene interactions across two pathways

	Oracle	Uni. Sel.	Ordinary Lasso	TS-GSIS Lasso	OGS Ridge	OGS Lasso
censoring rate = 50%						
RMSE.M	0.064	0.067	0.067	0.067	0.069	0.067
T.model	1	0	0	0	0.172	0
Tint.model	1	0	0.098	0.064	0.172	0.078
Sen.	1	0.200	0.504	0.586	0.977	0.659
Spe.	1	1	0.999	0.999	0.767	0.999
S.model	84	18.529	137.623	140.039	24,970	145.250
Deviance	−136.378	−38.797	−57.657	−71.756	−11.113	−83.105
* c*-index	0.928	0.765	0.838	0.856	0.600	0.875
censoring rate = 65%						
RMSE.M	0.063	0.067	0.068	0.067	0.069	0.067
T.model	1	0	0	0	0.279	0
Tint.model	1	0	0.051	0.051	0.279	0.084
Sen.	1	0.180	0.435	0.519	0.982	0.573
Spe.	1	1	0.999	0.999	0.756	0.999
S.model	84	17.284	127.991	130.405	26,132	137.153
Deviance	− 124.043	−30.843	−44.032	−55.482	−5.697	−62.328
* c*-index	0.936	0.761	0.822	0.845	0.598	0.860

**Table 8:** Results of Simulation 2 (3): The performances of OGS compared with other approaches under coexistence of within- and between-pathway gene-gene interactions

	Oracle	Uni. Sel.	Ordinary Lasso	TS-GSIS Lasso	OGS Ridge	OGS Lasso
censoring rate = 50%						
RMSE.M	0.068	0.071	0.071	0.070	0.072	0,070
T.model	1	0	0	0	0.045	0
Tint.model	1	0	0.085	0.030	0.045	0.035
Sen.	1	0.185	0.533	0.575	0.955	0.632
Spe.	1	1	0.999	0.999	0.763	0.999
S.model	87	17.625	147.060	137.595	25,425	146.765
Deviance	−135.986	−38.636	−65.861	−73.924	−5.523	−83.062
* c*-index	0.916	0.751	0.839	0.845	0.587	0.859
censoring rate = 65%						
RMSE.M	0.067	0.071	0.071	0.071	0.072	0.070
T.model	1	0	0	0	0.104	0
Tint.model	1	0	0.010	0.035	0.104	0.050
Sen.	1	0.177	0.464	0.518	0.961	0.582
Spe.	1	1	0.999	0.999	0.759	0.999
S.model	87	17.094	134.752	133.153	25,793	139.218
Deviance	−128.426	−33.808	−52.046	−60.786	−7.674	−70.564
* c*-index	0.925	0.752	0.826	0.837	0.601	0.855

### The DLBCL analysis

The DLBCL data [24] contain two sets of gene expression data, CHOP and R-CHOP. The CHOP dataset is under a combination chemotherapy with cyclophosphamide, doxorubicin, vincristine and prednisone; R-CHOP is under the current golden standard treatment, the rituxima immunotherapy in addition to the chematherapy in CHOP. The CHOP and R-CHOP datasets consist of censored survival outcomes from 181 and 233 patients, respectively, with gene expression data from the same 3833 genes after the filtering process. The censoring rates are 42% and 74% in the CHOP and R-CHOP datasets, respectively. These two microarray datasets can be downloaded from the R package “*bujar*” [25]. In our analysis, we divide randomly the patients into 207:207 training/test datasets from the pool of R-CHOP and CHOP datasets. There were no significant differences in clinical survival outcome between subjects in the two datasets.

We apply the proposed OGS approach to the DLBCL data with the prior pathway information obtained from the KEGG pathway database. The following analysis is based on the 451 genes mapped into 165 pathways in the DLBCL data, which result in 101,926 main and two-way interaction covariates.

In Steps 1–3 of the OGS approach, we identify 6 significant pathways and 2 significant cross-talk pathway interactions. In Step 4 of the OGS method, the Cox’s model with the Ridge or Lasso penalty is applied to the training data to establish the final prediction model. In particular, the OGS method with the Lasso penalty leads to a prediction model with 5 main and 10 two-way interaction covariates. The “Univariate Selection” and “Ordinary Lasso” methods are applied directly to the whole 101,926 covariates in the training data to build the prediction models. The “Overlap Lasso” method is obtained by applying the R package “grpregOverlap” [6], which performs group selection among overlapping groups with the Lasso penalty but without considering interactions among features.

**Fig. 4:**
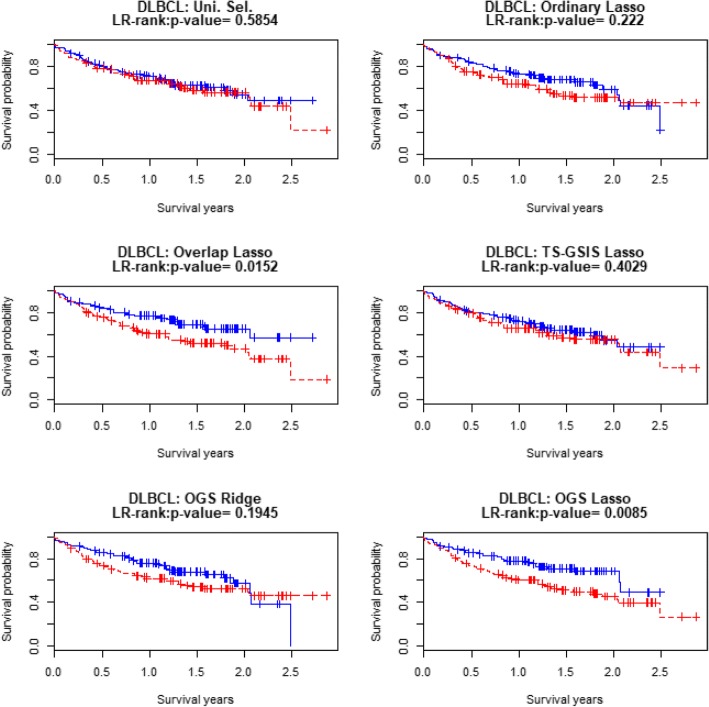
Kaplan-Meier curves for the 207 subjects in the DLBCL with the testing data. Good (blue) and poor (red) groups are identified by the median of the PI’s in the testing dataset

Table 9 displays several survival prediction accuracy measures for different approaches in the test data. We see that the OGS method with the Lasso penalty has better performances compared to existing methods in the test data. Fig. 4 displays the Kaplan-Meier survival curves for the “good” (blue) and “poor” (red) prognosis groups in the test data, which are formed according to whether the prognosis index (PI) value is lower or higher than the median PI value (see the Results section for detail). It is seen that that the two survival curves are better separated by the OGS approach than by the existing methods.

**Table 9:** Results of prediction accuracies of different methods based on DLBCL data

	Uni. Sel.	Ordinary Lasso	Overlap Lasso	TS-GSIS Lasso	OGS Ridge	OGS Lasso
Cox-test	0.8173	0.4487	0.0087	0.1102	0.3828	0.0007
LR-test	0.5854	0.2220	0.0152	0.4029	0.1945	0.0085
Deviance	183.1428	−0.4282	−6.4363	−1.9859	2.3566	−10.6504
*c*-index	0.5136	0.5367	0.5842	0.5468	0.5568	0.6001

In DLBCL data, we discard 3382 genes that are not mapped into any pathways in the KEGG pathway database based on the latent effect approach. We also perform the other OGS analysis putting the 3382 ungrouped genes together as an additional group. The results from such an analysis are similar to those presented here.

### The NSCLC analysis

The NSCLC data of Chen et al. [14] is available from NCBI with accession number GSE4882. The data contain censored survival outcomes from 125 lung cancer patients and their gene expression profiles for 672 genes. The censoring rate is 65%. Following Emura et al. [13], we consider the subset consisting of 485 genes, and, following Chen et al. [14], we divide the patients into 63:62 training/test datasets.

Based on the GO biological process database, prior pathway information for 251 genes mapped into 344 pathways are utilized, which lead to a total number of 31,626 main and two-way interaction covariates. Using the OGS approach, we identify 2 significant pathways but no significant pathway interaction, and the final prediction model obtained by the Lasso method includes main effects from two genes, DUSP6 and LCK. Indeed, the two genes are also included in the five-gene signature by Chen et al. [14], and are found to be strongly associated with lung cancer in other literatures ([26–28] and so on).

**Fig. 5:**
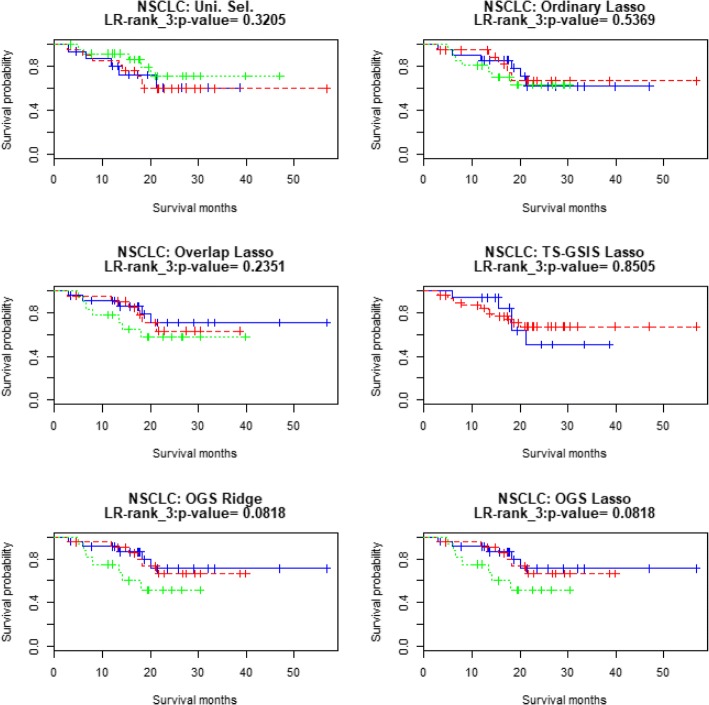
Kaplan-Meier curves for the 62 subjects in the NSCLC testing data. Good (blue), medium (red) and poor (green) groups are identified by the tertile of the PI’s in the test dataset

**Fig. 6:**
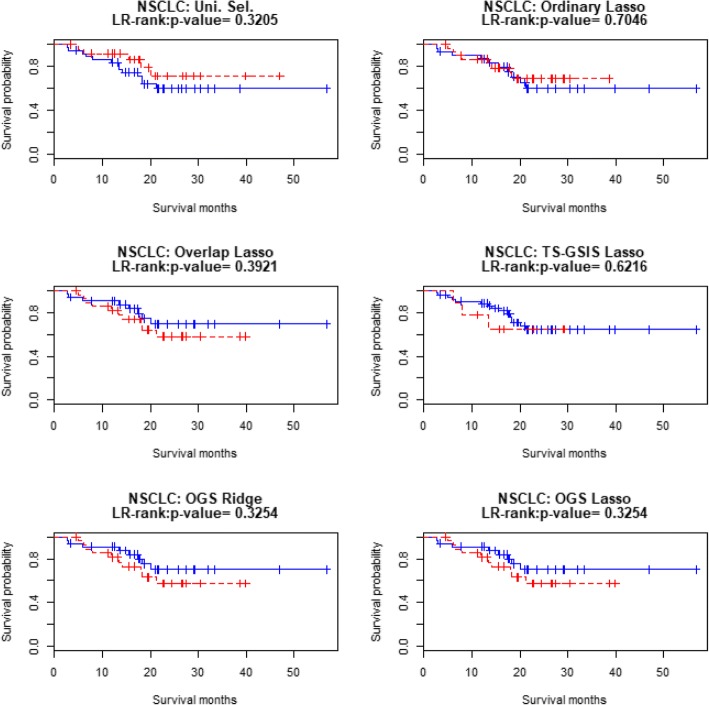
Kaplan-Meier curves for the 62 subjects in the NSCLC testing data. Good (blue) and poor (red) groups are identified by the median of the PI’s in the test dataset

Table 10 shows the prediction accuracy measures for patients’ survival in the test sample of the NSCLC data, where the measure LR-test_3 is the *p*-value of the log-rank test for equality of survival distributions among the three prognosis groups divided by the tertiles of the PI values in the test sample. Fig. 5 displays the three Kaplan-Meier survival curves for three prognosis groups (“good”, “medium”, “poor” groups according tertiles of the PI values) in the test sample of the NSCLC data (in this case the LR-test for the two prognosis groups divided by the median PI is less significant. Fig. 6 displays the two Kaplan-Meier survival curves for the two prognosis groups). In all these measures, the OGS method with the Lasso penalty performs better than the Ordinary Lasso.

**Table 10:** Results of prediction accuracies of different methods based on NSCLC data (using the training and test sets as in Chen et al. [14])

	Uni. Sel.	Ordinary Lasso	Overlap Lasso	TS-GSIS Lasso	OGS Ridge	OGS Lasso
Cox-test	0.8381	0.6215	0.3441	0.8467	0.2372	0.2484
LR-test	0.3205	0.7046	0.3921	0.6216	0.3254	0.3254
Deviance	40.1323	0.4820	−0.3605	3.9135	−1.3311	−1.0551
*c*-index	0.4485	0.5565	0.5775	0.5394	0.5966	0.5966
LR-test_3	0.3205	0.5369	0.2351	0.8505	0.0818	0.0818

Besides, we also apply the 10-fold cross-validation method to evaluate the performance of the OGS method for survival prediction in the NSCLC data. In the 10-fold cross-validation process, most of the time the OGS still identifies the same prediction model containing the main effects of DUSP6 and LCK genes. Table 11 shows the performances of the OGS method in the NSCLC data with the performance evaluation based on the 10-fold cross-validation, i.e., the average of the results among 10 folds. We see that the performance patterns are similar to those in Table 10, and the OGS with the Lasso penalty still outperforms the other methods.

**Table 11:** Results of prediction accuracies of different methods based on NSCLC data with 10-fold cross-validation procedure

	Uni. Sel.	Ordinary Lasso	Overlap Lasso	TS-GSIS Lasso	OGS Ridge	OGS Lasso
Cox-test	0.1688	0.7781	0.1734	0.4678	0.1435	0.1426
LR-test	0.6795	0.5696	0.1120	0.5337	0.8289	0.4356
Deviance	22.4633	1.5506	−0.1409	−0.5405	−1.0941	−1.4853
*c*-index	0.7273	0.3333	0.6970	0.6061	0.6235	0.7576
LR-test_3	0.1997	0.1990	0.1194	0.1053	0.1150	0.1085

In NSCLC data, we discard 234 genes that are not mapped into any pathways in the GO biological process database based on the latent effect approach. The OGS approach for putting the 234 ungrouped genes together as an additional group results in the same prediction model as the one presented above.

## Discussion

The OGS procedure can further adjust for confounding covariates (e.g. environmental factors) when all the models involved, including the null model without using gene expression covariate data, further adjust for the confounding variables; see [9, 10] for the SKAT statistics with confounding covariates for quantitative, qualitative and survival traits.

In this article, we consider two-way and multiplicative interactions as a simple way to implement interaction assessments. Examination of higher-order and general forms of interaction is challenging and deserves further research. Besides, the OGS method employs the latent effect approach to deal with the overlapping structure among pathways. This approach requires the gene grouping (pathway) structure to be pre-specified and is restricted to genes that can be assigned to at least one group (pathway). It is interesting to study how these restrictions can be relaxed to improve the performances of gene selection and survival prediction. Yu and Liu [29] propose a procedure for sparse regression incorporating a comprehensive graphical structure (SRIG) among predictors, and we would like to extend the current proposal by employing the SRIG approach.

The idea of group screening procedure we propose can also be applied to detect gene-environment interactions. In the first step, we still apply the overlapping group method to identify the causal pathways ${\hat{\mathrm{M}}}_{main}$. In the second step, we apply the SKAT test to obtain the group-specific significance, where each of the groups are formed by the interactions between one gene from each of the causal pathways in ${\hat{\mathrm{M}}}_{main}$ and one environment factor in Z, where Z is the set of environment covariates whose interactions with genes are of interest. In step 3, we select significant gene-environment interactions, where the permutation procedure and the cutoff determination are the same as those in the original OGS, except that now the permeation is applied to the covariate matrix consisting of both gene and environmental covariates. Finally, the penalized regression with the Ridge or Lasso penalty is still applied to build the final prediction model based on the genes in ${\hat{\mathrm{M}}}_{main}$, the environmental covariates, and the selected gene-environment interactions. We plan to study extensions of the OGS method, including the extension to gene-environment interactions, in our future research.

In this work we focus on survival prediction based on the Cox’s proportional hazards model. In the case where the proportional hazards assumption is not appropriate, an alternative model, such as the proportional odds model, that proves to be appropriate can be used instead in the OGS procedure proposed. The required modification with models alternative to the proportional hazards is quite straightforward. For example, the SKAT statistic involved in OGS can be simply modified by using residuals from the alternative model considered.

## Conclusions

It has been a long-lasting interest in the bioinformatics field for detecting the pairwise gene-gene interactions. In this paper we propose an overlapping group screening procedure to identify causal genes and gene-gene interactions efficiently by incorporating prior pathway information, where the pathways involved are allowed to overlap one another. Specifically, we utilize the gene pathway information via the latent effect approach which formally accounts for the possibly overlapping grouping structure. In addition, we utilize the SKAT testing approach to perform powerful screening of main and interaction effects. Simulation and real data studies demonstrate that the new proposal can substantially improve the accuracy of gene and gene-gene interaction selection and hence lead to more accurate survival prediction compared with the common analyses that ignore the pathway information. We provide an R package “*OGS*” to perform Steps 1–3 of the proposed OGS method, together with the reference manual describing how to perform “*OGS*” and the code used in our simulation. Please see Additional Files 2 and 3 for detail.

The OGS approach is general in that they can accommodate various types of clinical outcomes and regression models, such as quantitative, qualitative, and survival outcomes modeled by linear, logistic, and Cox’s regression models, respectively. In the paper the OGS approach based on the Cox’s model for gene selection, effect estimation, and survival outcome prediction has been examined. The OGS methods for continuous and binary outcomes based respectively on the linear and logistic regression models are discussed in Additional File 1. The extension of OGS to more flexible models, such as those based on the kernel methods [30], deserves further research and will be studied in our future work.

The importance of gene-gene interactions have been discussed widely in literature. For example, Cordell [31] discussed the need of considering gene-gene interactions in genetic studies of complex diseases. Fang et al. [2] identified and confirmed important gene-gene interactions related to rheumatoid arthritis. We believe that the proposed overlapping group screening (OGS) approach provides an useful tool to this important task in delineating the underlying disease etiology.

## Additional files

Additional file 1: The full detail and performances of the OGS approach for survival, continuous and binary outcomes, and settings where some of genes are shared by three groups (pathways). (DOC 317 kb)

Additional file 2: An R package “*OGS*”, which is a Windows binaries zip file. (ZIP 29 kb)

Additional file 3: A reference manual for the “*OGS*” package. (PDF 77 kb)

## Abbreviations

DLBCL: Diffuse large B-cell lymphoma
Lasso: Least absolute shrinkage and selection operator
LR-test: Log-rank test
NSCLC: Non-small-cell lung cancer
OGS: Overlapping group screening
PI: Predictor index
RMSE: Root mean squared error
SKAT: Sequence kernel association test
SNPs: Single-nucleotide polymorphisms
SRIG: sparse regression incorporating graphical structure
SVM: Support vector machine
TS-GSIS: Two-stage grouped sure independence screening
TS-SIS: Two-stage sure independence screening
