# Supplementary material for: Overlapping group screening for detection of gene-gene interactions: application to gene expression profiles with survival trait
Source: BMC Bioinformatics. 2018 Sep 21;19:335. doi: 10.1186/s12859-018-2372-2 (PMC6150983; doi:10.1186/s12859-018-2372-2)
Supplement: Supplementary file 1 — The full detail and performances of the OGS approach for survival, continuous and binary outcomes, and settings where some of genes are shared by three groups (pathways). (DOC 317 kb) [file 12859_2018_2372_MOESM1_ESM.doc]

Web-based Supplementary Materials for

” **Overlapping** **Group Screening for Detection of Gene-gene Interactions: Application to Gene Expression Profiles with Survival Trait**”

by

Jie-Huei Wang and Yi-Hau Chen

S.1 Supplement to the performance of OGS approach for Cox’s regression model:

We consider survival data with a cohort size 500 as the training set, where each subject’s survival time follows the Cox’s proportional hazards model

$\lambda_{0}\left(\left.t\right|\mathbf{x}\right)=0.1\cdot\exp\left(\mathbf{x}^{/}\mathbf{\beta}\right)$,

with $\mathbf{\beta}$ measuring the log-relative risk with respect to the covariates and the covariates $\mathbf{x}$ jointly following a multivariate standard normal distribution with correlation $corr\left(x_{\cdot j},x_{\cdot k}\right)=0.5^{\left|j-k\right|}$. The censoring time distribution follows a uniform $U(0,1)$ distribution. We then generate survival data, independent of the training data, with a cohort of size 100 as the test data to assess the prediction accuracy for different methods.

**Simulation Setting 1:** **equal group size da****ta**

In this simulation study, the design matrix consists of 15 groups with each group having 10 members. The group size (i.e. the number of genes in each pathway) and the overlapping structure (i.e. the number of shared genes between two overlapping pathways) are shown below.

**Table S1** Data structure in Simulation 1

| Pathway | 1 | 2 | 3 | 4 | 5 | 6 | 7 | 8 | 9 | 10 | 11 | 12 | 13 | 14 | 15 |
| --- | --- | --- | --- | --- | --- | --- | --- | --- | --- | --- | --- | --- | --- | --- | --- |
| Gene Size | 10 | 10 | 10 | 10 | 10 | 10 | 10 | 10 | 10 | 10 | 10 | 10 | 10 | 10 | 10 |
| Overlapping | 3 3 3 3 3 3 3 3 3 3 3 3 3 3 | | | | | | | | | | | | | | |

For example, group 1 contains 10 genes, as group 2 does, but the two groups contain only 17 unique genes, and 3 genes are shared between the two groups. As a result, there are a total of 108 genes (q=108) and 150 latent effects in this example. Fig. S1 shows the gene indices of the pathways in the data. Pathways 1, 4, 7, 10, and 13 are set to be effective, and genes in each of them have constant latent effects of 2, -2, 3, -3, and 4, respectively. In addition, interactions $\left(x_{\cdot1}\times x_{\cdot5},x_{\cdot2}\times x_{\cdot7},x_{\cdot3}\times x_{\cdot6}\right)$in pathway 1 and $\left(x_{\cdot22}\times x_{\cdot32},x_{\cdot23}\times x_{\cdot33},x_{\cdot24}\times x_{\cdot34}\right)$ between pathways 4 and 5 are set to be effective with effects $\left(v,1.5v,2v\right)$ and $\left(-v,-1.5v,-2v\right)$, respectively. The number of effective genes and gene-gene interaction pairs is 56 among the total 5,886 genes and gene-pairs. We examine different methods under various values of $v$. The censoring proportion for the survival outcome is around 50%.

**Simulation Setting 2: un****equal group size data**

In this simulation study, the design matrix consists of 15 groups with each group having different group sizes. The group size (number of genes in each pathway) and the overlapping structure (number of genes shared by two overlapping pathways) are shown below.

**Table S2** Data structure in Simulation 2

| Pathway | 1 | 2 | 3 | 4 | 5 | 6 | 7 | 8 | 9 | 10 | 11 | 12 | 13 | 14 | 15 |
| --- | --- | --- | --- | --- | --- | --- | --- | --- | --- | --- | --- | --- | --- | --- | --- |
| Gene Size | 3 | 3 | 3 | 6 | 6 | 6 | 9 | 9 | 9 | 15 | 15 | 15 | 24 | 24 | 24 |
| Overlapping | 1 1 2 2 3 3 5 5 8 8 | | | | | | | | | | | | | | |

For example, pathway 4 contains 6 genes, as group 5 does, and the two groups contain 10 unique genes, and 2 genes are shared by the two groups. As a result, there are 133 genes (q=133) and 171 latent effects in this example. Fig. S2 shows the gene indices of the pathways in this data. Pathways 1, 4, 7, 10, and 13 are effective, and genes in each of them have constant latent effects of 3, -2, 3, -2, and 2, respectively. Also, interactions $\left(x_{\cdot22}\times x_{\cdot23},x_{\cdot24}\times x_{\cdot25},x_{\cdot26}\times x_{\cdot27}\right)$in pathway 7 and $\left(x_{\cdot43}\times x_{\cdot60},x_{\cdot44}\times x_{\cdot61},x_{\cdot45}\times x_{\cdot62}\right)$ between pathways 10 and 11 are effective with effects $\left(v,1.5v,2v\right)$ and $\left(-v,-1.5v,-2v\right)$, respectively. The number of effective genes and gene-pair interactions is 63 among the total 8,911 genes and gene-pairs. We examine different methods under various values of $v$. The average censoring proportion for the survival outcome is 44%.

From the simulation results shown in Tables S3 and S4, the OGS method using the Lasso penalty outperforms the OGS method using the Ridge penalty. Also, compared to the existing methods, OGS with the Lasso penalty performs substantially better than the Univariate Selection and the TS-GSIS with the Lasso penalty methods in variable selection (T.model), estimation (RMSE.M), and prediction (Deviance, *c*-index). When the interaction effects are relatively weak ($v=3$), OGS with the Lasso penalty performs worse than or similarly to the ordinary Lasso method, while when the interaction effects are relatively strong ($v=4$ or 5), OGS with the Lasso penalty performs better than the ordinary Lasso in variable selection, estimation, and survival prediction.

**Table S3** The performances of OGS compared with other approaches in Simulation 1

|  | Oracle | Uni. Sel. | Ordinary  Lasso | TS-GSIS  Lasso | OGS  Ridge | OGS  Lasso |
| --- | --- | --- | --- | --- | --- | --- |
| v=3, censoring rate=52% | | | | | | |
| RMSE.M | 0.288 | 0.295 | 0.277 | 0.284 | 0.286 | 0.280 |
| T.model | 1 | 0 | 0.808 | 0.290 | 0.424 | 0.424 |
| S.model | 56 | 32.968 | 169.606 | 94.928 | 219.542 | 96.302 |
| Deviance | -137.669 | -84.192 | -190.378 | -144.630 | -141.101 | -161.612 |
| *c*-index | 0.936 | 0.857 | 0.966 | 0.928 | 0.925 | 0.940 |
| v=4, censoring rate=52% | | | | | | |
| RMSE.M | 0.315 | 0.322 | 0.302 | 0.227 | 0.305 | 0.223 |
| T.model | 1 | 0 | 0.798 | 0.618 | 0.754 | 0.754 |
| S.model | 56 | 34.882 | 171.882 | 114.636 | 249.164 | 116.268 |
| Deviance | -134.926 | -85.149 | -194.046 | -266.993 | -177.137 | -281.778 |
| *c*-index | 0.931 | 0.858 | 0.966 | 0.984 | 0.948 | 0.985 |
| v=5, censoring rate=51% | | | | | | |
| RMSE.M | 0.347 | 0.353 | 0.331 | 0.250 | 0.328 | 0.240 |
| T.model | 1 | 0 | 0.716 | 0.666 | 0.866 | 0.866 |
| S.model | 56 | 35.890 | 172.320 | 117.774 | 247.408 | 121.614 |
| Deviance | -131.673 | -84.395 | -196.076 | -272.325 | -196.928 | -285.778 |
| *c*-index | 0.930 | 0.857 | 0.969 | 0.984 | 0.959 | 0.986 |

**Table S4** The performances of OGS compared with other approaches in Simulation 2

|  | Oracle | Uni. Sel. | Ordinary  Lasso | TS-GSIS  Lasso | OGS  Ridge | OGS  Lasso |
| --- | --- | --- | --- | --- | --- | --- |
| v=3, censoring rate=46% | | | | | | |
| RMSE.M | 0.203 | 0.210 | 0.194 | 0.178 | 0.214 | 0.166 |
| T.model | 1 | 0 | 0.912 | 0.548 | 0.674 | 0.674 |
| S.model | 63 | 34.126 | 184.326 | 151.419 | 973.082 | 157.046 |
| Deviance | -139.722 | -75.066 | -198.654 | -246.622 | -49.942 | -267.153 |
| *c*-index | 0.921 | 0.823 | 0.961 | 0.971 | 0.831 | 0.979 |
| v=4, censoring rate=44% | | | | | | |
| RMSE.M | 0.229 | 0.234 | 0.216 | 0.184 | 0.239 | 0.178 |
| T.model | 1 | 0 | 0.924 | 0.834 | 0.956 | 0.956 |
| S.model | 63 | 33.548 | 187.998 | 180.772 | 1010.954 | 182.174 |
| Deviance | -134.691 | -71.659 | -215.963 | -285.914 | -51.828 | -298.168 |
| *c*-index | 0.912 | 0.813 | 0.964 | 0.979 | 0.826 | 0.981 |
| v=5, censoring rate=42% | | | | | | |
| RMSE.M | 0.258 | 0.263 | 0.241 | 0.204 | 0.268 | 0.197 |
| T.model | 1 | 0 | 0.944 | 0.796 | 0.964 | 0.964 |
| S.model | 63 | 30.720 | 190.698 | 182.397 | 998.648 | 187.702 |
| Deviance | -128.983 | -67.963 | -227.992 | -298.320 | -50.164 | -307.435 |
| *c*-index | 0.903 | 0.804 | 0.966 | 0.979 | 0.819 | 0.981 |

S.2 Supplement to the performance of OGS approach for linear regression model:

In this simulation study, we would like to examine the performance of OGS approach for linear regression model in gene selection, effect estimation and prediction accuracy. Furthermore, we also compare the performances of OGS with the common methods. The simulation study was based on data generated includes the distribution, effect size, interaction effect, overlapping structure of genes in exactly the same manner as those generated in Section S.1. The following numerical results are carried out using 500 replicates. The quantitative trait following the linear regression model is

$y_{i}=\mathbf{x}_{i}^{/}\mathbf{\beta}+\varepsilon_{i},i=1,\cdots,\mathrm{N}$, where $\varepsilon\sim N\left(0,1\right)$.

We note that the procedures of OGS in linear model are the same as the OGS in Cox’s model. These two differences compare to Cox’s model for SKAT statistic are residual term , where is the estimate of intercept term of the corresponding model and the element , where is the estimator of $\sigma$ of the corresponding model.

In evaluating criteria of linear model prediction, we report three measures for prediction accuracy:

1. A simple correlation between the true and predicted values.
2. Min_Max accuracy of a regression model is mean of the ratio of minimum of true and predicted values to maximum of true and predicted values.
3. Mean absolute percentage error (MAPE) of a regression model is mean of the absolute of the ratio of true minus predicted values to true values.

We note that the simple correlation and Min_Max accuracy close to 1, and smaller MAPE correspond to better prediction ability. The median values over 500 replications for these predicted measures are reported. Tables S3 and S4 indicate that The OGS Lasso perform better than the existing approaches in variable selection, estimation, and prediction.

**Table S5** The performances of OGS compare with the other approaches in equal group size data

|  | Oracle | Uni.  Sel. | Ordinary  Lasso | OGS  Ridge | OGS  Lasso |
| --- | --- | --- | --- | --- | --- |
| v=3 | | | | | |
| RMSE.M | 0.006 | 0.158 | 0.179 | 0.206 | 0.012 |
| T.model | 1 | 0 | 0 | 0.656 | 0.656 |
| S.model | 56 | 50.684 | 49.580 | 524.532 | 74.400 |
| Correlation | 1 | 0.945 | 0.920 | 0.906 | 0.999 |
| Min_Max  MAPE(%) | 1.003  11.600 | 0.912  112.916 | 0915  125.119 | 1.015  98.331 | 0.998  20.723 |
| v=4 | | | | | |
| RMSE.M | 0.006 | 0.159 | 0.222 | 0.245 | 0.012 |
| T.model | 1 | 0 | 0 | 0.836 | 0.836 |
| S.model | 56 | 53.982 | 49.304 | 526.404 | 64.236 |
| Correlation | 1 | 0.947 | 0.879 | 0.893 | 1 |
| Min_Max  MAPE(%) | 1.001  10.806 | 0.970  115.026 | 0.887  144.433 | 1.011  98.292 | 1.001  13.052 |
| v=5 | | | | | |
| RMSE.M | 0.006 | 0.168 | 0.270 | 0.276 | 0.013 |
| T.model | 1 | 0 | 0 | 0.864 | 0.864 |
| S.model | 56 | 56.910 | 48.900 | 540.320 | 61.734 |
| Correlation | 1 | 0.945 | 0.838 | 0.884 | 1 |
| Min_Max  MAPE(%) | 0.995  10.437 | 0.979  114.644 | 0.807  156.993 | 1.027  97.707 | 0.995  12.598 |

**Table S6** The performances of OGS compare with the other approaches in unequal group size data

|  | Oracle | Uni.  Sel. | Ordinary  Lasso | OGS  Ridge | OGS  Lasso |
| --- | --- | --- | --- | --- | --- |
| v=3 | | | | | |
| RMSE.M | 0.008 | 0.126 | 0.140 | 0.176 | 0.014 |
| T.model | 1 | 0 | 0 | 0.816 | 0.816 |
| S.model | 63 | 54.934 | 58.636 | 1704.386 | 112.414 |
| Correlation | 0.999 | 0.925 | 0.890 | 0.776 | 0.999 |
| Min_Max  MAPE(%) | 0.993  14.599 | 0.861  129.781 | 0.806  151.288 | 0.929  125.661 | 0.986  22.072 |
| v=4 | | | | | |
| RMSE.M | 0.008 | 0.137 | 0.179 | 0.201 | 0.016 |
| T.model | 1 | 0 | 0 | 0.938 | 0.938 |
| S.model | 63 | 56.576 | 57.798 | 1764.428 | 96.234 |
| Correlation | 0.999 | 0.919 | 0.837 | 0.763 | 0.998 |
| Min_Max  MAPE(%) | 0.990  14.912 | 0.847  137.505 | 0.759  173.294 | 0.921  131.949 | 0.985  22.803 |
| v=5 | | | | | |
| RMSE.M | 0.008 | 0.153 | 0.217 | 0.231 | 0.019 |
| T.model | 1 | 0 | 0 | 0.968 | 0.968 |
| S.model | 63 | 55.796 | 56.816 | 1839.050 | 86.750 |
| Correlation | 0.999 | 0.907 | 0.784 | 0.749 | 0.998 |
| Min_Max  MAPE(%) | 0.991  14.392 | 0.837  149.369 | 0.742  181.720 | 0.749  132.422 | 0.980  23.069 |

S.3 Supplement to the performance of OGS approach for logistic regression model:

In logistic regression data settings, the qualitative trait following the logistic regression model is

$\Pr\left(y_{i}=1|\mathbf{x}_{i}\right)=\frac{\exp\left(\mathbf{x}_{i}^{/}\mathbf{\beta}\right)}{1+\exp\left(\mathbf{x}_{i}^{/}\mathbf{\beta}\right)}$,

with the risk effect $\mathbf{\beta}$ respect to the covariates and the covariates $\mathbf{x}$ follow a uniform distribution $\text{U}\left(\text{-1},1\right)$. We generate the data with a cohort of size 1,000 and 200 as training and testing sets, respectively.

We also consider two datasets: equal and unequal group size datasets. These two overlapping structures of genes are the same as those produced in **Section S.1.**

In equal group size data, pathways 1 and 4 are set to be effective, and genes in each of them have constant latent effects of 1 and -1, respectively. In addition, interactions $\left(x_{\cdot1}\times x_{\cdot5},x_{\cdot2}\times x_{\cdot6}\right)$ in pathway 1 and $\left(x_{\cdot9}\times x_{\cdot22},x_{\cdot10}\times x_{\cdot23}\right)$ between pathways 1 and 4 are set to be effective with effects $\left(v,v\right)$ and $\left(-v,-v\right)$, respectively. The number of effective genes and gene-gene interaction pairs is 24 among the total number 5,886 of genes and gene-pairs.

In unequal group size data, pathways 1 and 4 are set to be effective, and genes in each of them have constant latent effects of 1 and -1, respectively. In addition, interactions $\left(x_{\cdot1}\times x_{\cdot2},x_{\cdot2}\times x_{\cdot3}\right)$ in pathway 1 and $\left(x_{\cdot2}\times x_{\cdot8},x_{\cdot3}\times x_{\cdot9}\right)$ between pathways 1 and 4 are set to be effective with effects $\left(v,v\right)$ and $\left(-v,-v\right)$, respectively. The number of effective genes and gene-gene interaction pairs is 13 among the total number 8,911 of genes and gene-pairs.

These two differences compare to Cox’s model for SKAT statistic are residual term , where is the estimate of intercept term of the corresponding model and the element , where . We then compute the predicted probability for each testing subject and set the probability threshold value as 0.5.

In evaluating criteria of binary classification prediction, we report three measures for prediction accuracy:

1. F-measure is the harmonic mean of precision and recall which is a traditional measure of a test’s accuracy.

2. AUC-ROC score: The Area Under an ROC Curve is used to determine model fit.

3. Positive likelihood ratio (LR+) is determined as the ratio of true positive rate to false positive rate.

We note that the higher F-measure, AUC-ROC score and LR+ correspond to better prediction ability. The median values over 500 replications for these predicted measures are reported. Tables S5 and S6 indicate that The OGS Lasso perform better than the existing approaches in variable selection, estimation, and prediction.

**Table S7** The performances of OGS compare with the other approaches in equal group size data

|  | Oracle | Uni.  Sel. | Ordinary  Lasso | OGS  Ridge | OGS  Lasso |
| --- | --- | --- | --- | --- | --- |
| v=2 | | | | | |
| RMSE.M | 0.026 | 0.057 | 0.070 | 0.068 | 0.058 |
| T.model | 1 | 0.002 | 0 | 0.972 | 0.972 |
| S.model | 24 | 18.100 | 26.716 | 611.814 | 134.594 |
| F-measure | 0.807 | 0.754 | 0.741 | 0.749 | 0.800 |
| Auc_Roc  LR+ | 0.809  4.288 | 0.752  3.034 | 0.741  2.901 | 0.746  2.960 | 0.800  4.021 |
| v=2.5 | | | | | |
| RMSE.M | 0.026 | 0.065 | 0.080 | 0.077 | 0.066 |
| T.model | 1 | 0 | 0 | 0.992 | 0.992 |
| S.model | 24 | 17.222 | 27.454 | 644.470 | 139.694 |
| F-measure | 0.816 | 0.753 | 0.734 | 0.754 | 0.808 |
| Auc_Roc  LR+ | 0.815  4.351 | 0.751  3.014 | 0.735  2.739 | 0.754  3.020 | 0.809  4.217 |
| v=3 | | | | | |
| RMSE.M | 0.026 | 0.075 | 0.091 | 0.087 | 0.075 |
| T.model | 1 | 0 | 0 | 0.992 | 0.990 |
| S.model | 24 | 15.494 | 27.584 | 636.574 | 139.348 |
| F-measure | 0.817 | 0.741 | 0.716 | 0.752 | 0.811 |
| Auc_Roc  LR+ | 0.817  4.467 | 0.742  2.895 | 0.716  2.522 | 0.751  2.983 | 0.810  4.214 |

**Table S8** The performances of OGS compare with the other approaches in unequal group size data

|  | Oracle | Uni.  Sel. | Ordinary  Lasso | OGS  Ridge | OGS  Lasso |
| --- | --- | --- | --- | --- | --- |
| v=2 | | | | | |
| RMSE.M | 0.015 | 0.033 | 0.049 | 0.042 | 0.038 |
| T.model | 1 | 0.378 | 0 | 0.990 | 0.990 |
| S.model | 13 | 12.646 | 10.682 | 253.114 | 63.494 |
| F-measure | 0.774 | 0.764 | 0.683 | 0.747 | 0.771 |
| Auc_Roc  LR+ | 0.773  3.376 | 0.764  3.168 | 0.681  2.148 | 0.745  2.906 | 0.770  3.323 |
| v=2.5 | | | | | |
| RMSE.M | 0.015 | 0.040 | 0.059 | 0.050 | 0.045 |
| T.model | 1 | 0.228 | 0 | 0.990 | 0.990 |
| S.model | 13 | 12.174 | 11.146 | 259.036 | 64.854 |
| F-measure | 0.785 | 0.771 | 0.673 | 0.755 | 0.781 |
| Auc_Roc  LR+ | 0.783  3.558 | 0.766  3.239 | 0.675  2.064 | 0.754  2.999 | 0.780  3.515 |
| v=3 | | | | | |
| RMSE.M | 0.015 | 0.048 | 0.069 | 0.058 | 0.053 |
| T.model | 1 | 0.088 | 0 | 0.992 | 0.992 |
| S.model | 13 | 11.421 | 11.538 | 273.351 | 66.359 |
| F-measure | 0.796 | 0.769 | 0.656 | 0.758 | 0.787 |
| Auc_Roc  LR+ | 0.795  3.890 | 0.767  3.253 | 0.657  1.913 | 0.755  3.097 | 0.790  3.648 |

*P1: 1 2 3 4 5 6 7 8 9 10*

*P2: 8 9 10 11 12 13 14 15 16 17*

*P3: 15 16 17 18 19 20 21 22 23 24*

*P4: 22 23 24 25 26 27 28 29 30 31*

*P5: 29 30 31 32 33 34 35 36 37 38*

*P6: 36 37 38 39 40 41 42 43 44 45*

*P7: 43 44 45 46 47 48 49 50 51 52*

*P8: 50 51 52 53 54 55 56 57 58 59*

*P9: 57 58 59 60 61 62 63 64 65 66*

*P10: 64 65 66 67 68 69 70 71 72 73*

*P11: 71 72 73 74 75 76 77 78 79 80*

*P12: 78 79 80 81 82 83 84 85 86 87*

*P13: 85 86 87 88 89 90 91 92 93 94*

*P14: 92 93 94 95 96 97 98 99 100 101*

*P15: 99 100 101 102 103 104 105 106 107 108*

**Fig. S1** The gene indices of the pathways considered in Simulation 1

*P1: 1 2 3*

*P2: 3 4 5*

*P3: 5 6 7*

*P4: 8 9 10 11 12 13*

*P5: 12 13 14 15 16 17*

*P6: 16 17 18 19 20 21*

*P7: 22 23 24 25 26 27 28 29 30*

*P8: 28 29 30 31 32 33 34 35 36*

*P9: 34 35 36 37 38 39 40 41 42*

*P10:43 44 45 46 47 48 49 50 51 52 53 54 55 56 57*

*P11:53 54 55 56 57 58 59 60 61 62 63 64 65 66 67*

*P12:63 64 65 66 67 68 69 70 71 72 73 74 75 76 77*

*P13:78 79 80 81 82 83 84 85 86 87 88 89 90 91 92 93 94 95 96 97 98 99 100 101*

*P14:94 95 96 97 98 99 100 101 102 103 104 105 106 107 108 109 110 111 112 113 114 115 116 117*

*P15:110 111 112 113 114 115 116 117 118 119 120 121 122 123 124 125 126 127 128 129 130 131 132 133*

**Fig. S2** The gene indices of the pathways considered in Simulation 2

S.4 Simulation results for settings where some of the genes are shared by three groups:

We have performed an addition set of simulation studies (Simulation A (1)-(3)) where some of the genes are shared by three groups; the simulation setups in Simulation A (1)-(3) are essentially the same as those in Simulation setting 1 (1)-(3) (Tables 3.1-3.3) in the main text, respectively, except that now the first three groups (pathways) share two common genes (gene id 14, 15). As we can see from the following results, the performances of the proposed OGS method in these settings are similar to those in the settings of Tables 3.1-3.3 of the main text.

**Table S9.1** Results of Simulation A (1): The performances of OGS compared with other approaches under gene-gene interactions within one pathway

|  | Oracle | Uni. Sel. | Ordinary  Lasso | TS-GSIS  Lasso | OGS  Ridge | OGS  Lasso | Group  Lasso |
| --- | --- | --- | --- | --- | --- | --- | --- |
| censoring rate=50% | | | | | | |  |
| RMSE.M | 0.423 | 0.426 | 0.354 | 0.348 | 0.436 | 0.335 | 0.392 |
| T.model  Tint.model  Sen.  Spe. | 1  1  1  1 | 0  0.045  0.780  0.999 | 1  1  1  0.953 | 0.575  0.575  0.971  0.966 | 0.580  0.580  0.972  0.762 | 0.580  0.580  0.972  0.972 | 0  0  0.933  0.999 |
| S.model | 45 | 37.67 | 197.625 | 154.515 | 822.735 | 136.595 | 44.5 |
| Deviance | -125.979 | -106.508 | -281.189 | -269.122 | -49.053 | -279.436 | -173.805 |
| *c*-index | 0.923 | 0.892 | 0.984 | 0.981 | 0.855 | 0.984 | 0.930 |
| censoring rate=65% | | | | | | |  |
| RMSE.M | 0.42 | 0.424 | 0.377 | 0.368 | 0.436 | 0.356 | 0.391 |
| T.model  Tint.model  Sen.  Spe. | 1  1  1  1 | 0  0.055  0.762  0.999 | 1  1  1  0.962 | 0.690  0.690  0.978  0.968 | 0.700  0.700  0.980  0.671 | 0.700  0.700  0.978  0.970 | 0  0  0.933  0.999 |
| S.model | 45 | 37.60 | 170.995 | 148.680 | 1121.14 | 142.110 | 44.980 |
| Deviance | -127.209 | -105.698 | -234.657 | -232.452 | -44.893 | -244.663 | -157.625 |
| *c*-index | 0.935 | 0.902 | 0.982 | 0.983 | 0.839 | 0.985 | 0.936 |

**Table S9.2** Results of Simulation A (2): The performances of OGS compared with other approaches under gene-gene interactions across two pathways

|  | Oracle | Uni. Sel. | Ordinary  Lasso | TS-GSIS  Lasso | OGS  Ridge | OGS  Lasso | Group Lasso |
| --- | --- | --- | --- | --- | --- | --- | --- |
| censoring rate=50% | | | | | | |  |
| RMSE.M | 0.417 | 0.423 | 0.382 | 0.365 | 0.436 | 0.353 | 0.398 |
| T.model  Tint.model  Sen.  Spe. | 1  1  1  1 | 0  0.055  0.747  0.999 | 1  1  1  0.963 | 0.875  0.875  0.990  0.965 | 0.885  0.885  0.989  0.639 | 0.885  0.885  0.988  0.967 | 0  0  0.930  0.998 |
| S.model | 45 | 36.790 | 165.675 | 158.770 | 1227 | 153.365 | 47.830 |
| Deviance | -134.871 | -103.189 | -221.447 | -239.252 | -42.456 | -247.276 | -139.953 |
| *c*-index | 0.944 | 0.902 | 0.981 | 0.984 | 0.835 | 0.985 | 0.931 |
| censoring rate=65% | | | | | | |  |
| RMSE.M | 0.414 | 0.422 | 0.398 | 0.390 | 0.436 | 0.385 | 0.397 |
| T.model  Tint.model  Sen.  Spe. | 1  1  1  1 | 0  0.020  0.726  0.999 | 1  1  1  0.968 | 0.920  0.920  0.992  0.970 | 0.930  0.930  0.995  0.509 | 0.930  0.930  0.994  0.970 | 0  0  0.933  0.998 |
| S.model | 45 | 36.155 | 149.465 | 143.035 | 1651.83 | 141.360 | 49.075 |
| Deviance | -127.083 | -95.594 | -173.442 | -185.253 | -38.897 | -192.108 | -126.099 |
| *c*-index | 0.948 | 0.903 | 0.974 | 0.979 | 0.822 | 0.980 | 0.935 |

**Table S9.3** Results of Simulation A (3): The performances of OGS compared with other approaches under coexistence of within- and between-pathway gene-gene interactions

|  | Oracle | Uni. Sel. | Ordinary  Lasso | TS-GSIS  Lasso | OGS  Ridge | OGS  Lasso | Group Lasso |
| --- | --- | --- | --- | --- | --- | --- | --- |
| censoring rate=50% | | | | | | |  |
| RMSE.M | 0.458 | 0.463 | 0.399 | 0.406 | 0.471 | 0.391 | 0.446 |
| T.model  Tint.model  Sen.  Spe. | 1  1  1  1 | 0  0.005  0.703  0.999 | 1  1  1  0.956 | 0.510  0.510  0.962  0.967 | 0.535  0.535  0.968  0.673 | 0.535  0.535  0.965  0.969 | 0  0  0.875  0.999 |
| S.model | 48 | 36.865 | 191.295 | 155.290 | 1117.90 | 148.900 | 45.875 |
| Deviance | -127.866 | -94.368 | -269.084 | -237.856 | -46.217 | -261.862 | -130.552 |
| *c*-index | 0.923 | 0.869 | 0.982 | 0.973 | 0.825 | 0.977 | 0.897 |
| censoring rate=65% | | | | | | |  |
| RMSE.M | 0.455 | 0.461 | 0.418 | 0.417 | 0.471 | 0.410 | 0.445 |
| T.model  Tint.model  Sen.  Spe. | 1  1  1  1 | 0  0  0.699  0.999 | 1  1  1  0.964 | 0.615  0.615  0.968  0.969 | 0.625  0.625  0.974  0.619 | 0.625  0.625  0.970  0.971 | 0  0  0.875  0.999 |
| S.model | 48 | 37.185 | 167.350 | 147.570 | 1294.55 | 140.525 | 45.960 |
| Deviance | -122.638 | -92.018 | -217.516 | -209.662 | -41.813 | -219.586 | -115.690 |
| *c*-index | 0.930 | 0.880 | 0.980 | 0.977 | 0.820 | 0.981 | 0.903 |
