# Supplementary material for: Overlapping group screening for detection of gene-gene interactions: application to gene expression profiles with survival trait
Source: BMC Bioinformatics. 2018 Sep 21;19:335. doi: 10.1186/s12859-018-2372-2 (PMC6150983; doi:10.1186/s12859-018-2372-2)
Supplement: Supplementary file 2 — An R package “OGS”, which is a Windows binaries zip file. (ZIP 29 kb) [file 12859_2018_2372_MOESM2_ESM.zip › OGS/html/00Index.html]

R: Overlapping group screening (OGS) approach for detection of
gene-gene interactions

# Overlapping group screening (OGS) approach for detection of gene-gene interactions

---

## Documentation for package ‘OGS’ version 0.1

- DESCRIPTION file.

## Help Pages

|  |  |
| --- | --- |
| OGS | Overlapping group screening approach for detection of gene-gene interactions |
