# Supplementary material for: Overlapping group screening for detection of gene-gene interactions: application to gene expression profiles with survival trait
Source: BMC Bioinformatics. 2018 Sep 21;19:335. doi: 10.1186/s12859-018-2372-2 (PMC6150983; doi:10.1186/s12859-018-2372-2)
Supplement: Supplementary file 2 — An R package “OGS”, which is a Windows binaries zip file. (ZIP 29 kb) [file 12859_2018_2372_MOESM2_ESM.zip › OGS/html/OGS.html]

R: Overlapping group screening approach for detection of...

|  |  |
| --- | --- |
| OGS {OGS} | R Documentation |

## Overlapping group screening approach for detection of gene-gene interactions

### Description

The `OGS` function is used to compute the steps 1-3 of OGS approach, as described in Wang and Chen. (2018).

### Usage

```
OGS(Z, T, Group, family, main.penalty, main.nlambda, ridge.nlambda,
  ridge.nfolds, seed, standarize, character)
```

### Arguments

|  |  |
| --- | --- |
| `Z` | The gene expression profiles matrix, without an intercept. The column name of `Z` need to be defined if `character` is "T". The description of `character` cen be seen below. |
| `T` | The response types. For linear regression model, `T` is a quantitative trait. For logistic regression model, `T` is a qualitative trait. For Cox's regression model, `T` is the time-to-event outcome- a two-column matrix, the first column is the observable times (right censoring) which are time-to-event or censoring times and without ties at the event times, the second column is the status which is a binary variable with 1 indicating the event has occured and 0 indicating right censoring. |
| `Group` | The groups must be a list of vectos here, each containing integer indices or character names of gene features in the groups. Specific pathways database with gene lists can be inputted directly. Note that variables are not belong to the groups will be disgarded. |
| `family` | Either "cox", "gaussian", or "binomial", depending on the response `T`. |
| `main.penalty` | We call `grpregOverlap` function of `greregOverlap` package to do this procedure, the action of `main.penalty` is the same as that of `penalty` of `grpregOverlap` function. It is the first step of OGS approach. |
| `main.nlambda` | We call `grpregOverlap` function of `greregOverlap` package to do this procedure, the action of `main.nlambda` is the same as that of `nlambda` of `grpregOverlap` function. It is the first step of OGS approach. |
| `ridge.nlambda` | The number of lambda values for the ridge penalty, which is used to compute the weights of gene features for SKAT statistic. We call `glmnet` package to do this procedure. It is the second step of OGS approach. |
| `ridge.nfolds` | The tuning parameter of ridge penalty is estimated by k-folds cross-validation. k is `ridge.nfolds`. It is the second step of OGS approach. |
| `seed` | The `seed` of the random number generator to obtain reproducible results. The `seed` is used to permute randomly the original biomarkers among subjects to decouple the association between the biomarker and outcome data. It is the third step of OGS approach. |
| `standarize` | Setting to "F", point out the original gene espressions profiles are not standarized. We are going to standarize the gene features automatically by `OGS` function. Setting to "T", point out the original gene espressions profiles have been standarized. We maintain the original gene features matrix to do the following OGS approach. |
| `character` | Setting to "T", point out character names of gene features in the group; Setting to "F", point out integer indices of gene features in the group. |

### Value

Returns a list with components

|  |  |
| --- | --- |
| `main.p` | The causal pathways are selected by the R package `grpregOverlap`, which is the first step of OGS approach. |
| `int.p` | The causal pathway interaction groups determined by OGS approach. |
| `fz` | The final gene expression profiles matrix, they have been standarized. Some gene features are disgarded behind the latent effect approach. The column name of `fz` is gene symbol (`character`="T") or gene index (`character`="F"). |
| `allmodel` | The pool of the candidate model from the causal pathways and pathway interaction groups. The column name of `allmodel` is gene index, in which "Ga" means the ath main gene and "Ga&Gb" means the ath main gene interact with the bth main gene. The order of main gene based on the sequence of `fz` matrix. |
| `np` | The unselected pathways which are not mapped by genes. |

### Note

The missing value (NA) in the DATA is not allowed in this version.

### References

Wang JH, Chen YH (2018) Overlapping Group Screening for Detection of Gene-gene Interactions: Application to Gene Expression Profiles with Survival Trait. Accepted by BMC Bioinformatics.

### Examples

```
##### Simulation I #####
set.seed(5555)
library(CompQuadForm)
library(glmnet)
library(grpregOverlap)
library(stats)
library(survival)
library(utils)
tt=function(x){xx=x[,1]*x[,2]}
tg1=133; k1=1; k2=2; N=1000
group1=list(gr1=as.numeric(c(1:3)), gr2=as.numeric(c(3:5)), gr3=as.numeric(c(5:7)),
gr4=as.numeric(c(8:13)), gr5=as.numeric(c(12:17)), gr6=as.numeric(c(16:21)),
gr7=as.numeric(c(22:30)), gr8=as.numeric(c(28:36)), gr9=as.numeric(c(34:42)),
gr10=as.numeric(c(43:57)), gr11=as.numeric(c(53:67)), gr12=as.numeric(c(63:77)),
gr13=as.numeric(c(78:101)), gr14=as.numeric(c(94:117)), gr15=as.numeric(c(110:133)))
beta.latent.T1=c(c(rep(1.5*k1,3)), c(rep(0,3)), c(rep(0,3)),
c(rep(-k1,6)), c(rep(0,6)), c(rep(0,6)),
c(rep(0,9)), c(rep(0,9)), c(rep(0,9)),
c(rep(0,15)), c(rep(0,15)), c(rep(0,15)),
c(rep(0,24)), c(rep(0,24)), c(rep(0,24)))   # main group 1 and 4
Z1=matrix(runif(N*tg1,-1,1),N,tg1)
Z1.latent=expandX(Z1,group1)
int1=c(1,2); intZ1=as.matrix(tt(Z1[,int1])) # int group 1*1
int2=c(2,3); intZ2=as.matrix(tt(Z1[,int2])) # int group 1*1
int3=c(2,8); intZ3=as.matrix(tt(Z1[,int3])) # int group 1*4
int4=c(3,9); intZ4=as.matrix(tt(Z1[,int4])) # int group 1*4
INT=cbind(intZ1,intZ2,intZ3,intZ4)
inteffect=c(k2,k2,k2,k2)
XZ1.latent=as.matrix(cbind(Z1.latent,INT)); beta.latent.XT1=as.matrix(c(beta.latent.T1,inteffect))

#####  Cox's Regression Model for simulation I #####
lambda=0.1; C=matrix(runif(N,0,1),N,1); T1=X=S=matrix(0,N,1)
T1=log(runif(N,0,1))/(-lambda*exp(XZ1.latent%*%beta.latent.XT1)); X=pmin(T1,C); S=(T1==X)
OGStest1=OGS(Z=Z1, T=as.matrix(cbind(X,S)), Group=group1, family="cox", main.penalty="grLasso", 
main.nlambda=100, ridge.nlambda=100, ridge.nfolds=5, seed=555, standarize="F", character="F")

#####  Linear Rrgression Model for simulation I #####
T2=(XZ1.latent%*%beta.latent.XT1)+rnorm(N)
OGStest2=OGS(Z=Z1, T=T2, Group=group1, family="gaussian", main.penalty="grLasso", 
main.nlambda=100, ridge.nlambda=100, ridge.nfolds=5, seed=555, standarize="F", character="F")

#####  Logistic Rrgression Model for simulation I #####
pr=(exp(XZ1.latent%*%beta.latent.XT1))/(1+(exp(XZ1.latent%*%beta.latent.XT1)))
T3=rbinom(N,1,pr) 
OGStest3=OGS(Z=Z1, T=T3, Group=group1, family="binomial", main.penalty="grLasso", 
main.nlambda=100, ridge.nlambda=50, ridge.nfolds=5, seed=555, standarize="F", character="F")

##### Simulation II (not running) ######
#set.seed(5555)
#library(CompQuadForm)
#library(glmnet)
#library(grpregOverlap)
#library(MASS) # in order to generate multivariate normal distribution variable 
#library(stats)
#library(survival)
#library(utils)
#tt=function(x){xx=x[,1]*x[,2]}
#tg1=133; k1=2; k2=3; N=500
#group1=list(gr1=as.numeric(c(1:3)), gr2=as.numeric(c(3:5)), gr3=as.numeric(c(5:7)),
#gr4=as.numeric(c(8:13)), gr5=as.numeric(c(12:17)), gr6=as.numeric(c(16:21)),
#gr7=as.numeric(c(22:30)), gr8=as.numeric(c(28:36)), gr9=as.numeric(c(34:42)),
#gr10=as.numeric(c(43:57)), gr11=as.numeric(c(53:67)), gr12=as.numeric(c(63:77)),
#gr13=as.numeric(c(78:101)), gr14=as.numeric(c(94:117)), gr15=as.numeric(c(110:133)))
#beta.latent.T1=c( c(rep(1.5*k1,3)), c(rep(0,3)), c(rep(0,3)),
#c(rep(-k1,6)), c(rep(0,6)), c(rep(0,6)),
#c(rep(1.5*k1,9)), c(rep(0,9)), c(rep(0,9)),
#c(rep(-k1,15)), c(rep(0,15)), c(rep(0,15)),
#c(rep(k1,24)), c(rep(0,24)), c(rep(0,24)))    # main group 1, 4, 7, 10, 13
#Z1=matrix(0,N,tg1); beta0=matrix(0,tg1,1)
#sigma1=matrix(0,tg1,tg1)
#for (i in 1:tg1){
#for (j in 1:tg1){
#sigma1[i,j]=0.5^(abs(i-j))
#}}
#Z1=mvrnorm(N, beta0, sigma1, tol=1e-8, empirical=FALSE)
#Z1.latent=expandX(Z1,group1)
#int1=c(22,23); intZ1=as.matrix(tt(Z1[,int1])) # int group 7*7
#int2=c(24,25); intZ2=as.matrix(tt(Z1[,int2])) # int group 7*7
#int3=c(26,27); intZ3=as.matrix(tt(Z1[,int3])) # int group 7*7
#int4=c(43,60); intZ4=as.matrix(tt(Z1[,int4])) # int group 10*11
#int5=c(44,61); intZ5=as.matrix(tt(Z1[,int5])) # int group 10*11
#int6=c(45,62); intZ6=as.matrix(tt(Z1[,int6])) # int group 10*11
#INT=cbind(intZ1,intZ2,intZ3,intZ4,intZ5,intZ6)
#inteffect=c(k2,1.5*k2,2*k2,-k2,-1.5*k2,-2*k2)
#XZ1.latent=as.matrix(cbind(Z1.latent,INT)); beta.latent.XT1=as.matrix(c(beta.latent.T1,inteffect))

#####  Cox's Regression Model for simulation II  #####
#lambda=0.1; C=matrix(runif(N,0,1),N,1); T1=X=S=matrix(0,N,1)
#T1=log(runif(N,0,1))/(-lambda*exp(XZ1.latent%*%beta.latent.XT1)); X=pmin(T1,C); S=(T1==X)
#OGStest4=OGS(Z=Z1, T=as.matrix(cbind(X,S)), Group=group1, family="cox", main.penalty="grSCAD", 
#main.nlambda=100, ridge.nlambda=100, ridge.nfolds=5, seed=555, standarize="F", character="F")

#####  Linear Rrgression Model for simulation II  #####
#T2=(XZ1.latent%*%beta.latent.XT1)+rnorm(N)
#OGStest5=OGS(Z=Z1, T=T2, Group=group1, family="gaussian", main.penalty="grSCAD", 
#main.nlambda=100, ridge.nlambda=100, ridge.nfolds=5, seed=555, standarize="F", character="F")
```

---

[Package *OGS* version 0.1 Index]
